# Mutagenesis analysis of T380R mutation in the envelope protein of yellow fever virus

**DOI:** 10.1186/1743-422X-11-60

**Published:** 2014-03-29

**Authors:** Yan-Jang S Huang, John T Nuckols, Kate M Horne, Dana Vanlandingham, Mario Lobigs, Stephen Higgs

**Affiliations:** 1 Biosecurity Research Institute, Kansas State University, Manhattan, KS 66506, USA; 2 Department of Diagnostic Medicine and Pathobiology, College of Veterinary Medicine, Kansas State University, Manhattan, KS 66506, USA; 3 Medical Countermeasure Systems, Joint Vaccine Acquisition Program, Fort Detrick, MD 21702, USA; 4 Australian Infectious Disease Research Centre, The University of Queensland, St Lucia, QLD, Australia

**Keywords:** Yellow fever virus, 17D vaccine, *Aedes aegypti*

## Abstract

**Background:**

The RGD motif in the mosquito-borne flaviviruses envelope protein domain III (EDIII) FG loop was shown to bind negatively charged cellular molecules and mediate virus entry in mammals. However, its importance in virus entry in the mosquito has not yet been defined. The sequences of RGD motifs are conserved in JEV-serocomplex members primarily transmitted by *Culex* mosquitoes but absent from members of the DENV serocomplex, which utilize *Aedes* mosquitoes as vectors. Interestingly, the RGD sequence is present in the attenuated 17D strain of yellow fever virus as a result of the T380R mutation in the EDIII of Asibi strain following extensive *in vitro* passage in mice and chicken embryos and was found to contribute to the more rapid clearance in mice challenged with 17D. However, viral infectivity and dissemination in mosquitoes had not been evaluated for this mutant.

**Findings:**

The study utilized the reverse genetics system of YFV and *Ae. aegypti* RexD WE mosquitoes to assess the impact of a T380R mutation in YFV Asibi and 17D/Asibi M-E chimera. The T380R mutation led to higher infection rates but similar dissemination rates when introduced into the YFV Asibi strain and 17D/Asibi M-E chimera.

**Conclusions:**

While the increase of the positive charge in EDIII may reduce the virulence of YFV in mice, this mutation favored the establishment of the viral infection in *Ae. aegypti*. However, such gain in viral infectivity did not increase dissemination in infected mosquitoes.

## Findings

The transmission cycles of flaviviruses require successful utilization of cellular receptors in a wide variety of organisms including mammals, avians or arthropods. Although several molecules of mammalian origin have been proposed as putative receptors for mosquito-borne flaviviruses, knowledge about arthropod receptors remains limited [1]. Flavivirus receptor-binding activity in mammals was first shown to be mediated by the domain III of envelope protein (EDIII), eventually localized to the FG loop [2,3]. Although the sequences and structures of the FG loop among mosquito-borne and tick-borne flaviviruses are largely conserved, minor differences were still observed in the FG loop not only between both types of flaviviruses but also among mosquito-borne flaviviruses that utilize different vector species. Interestingly, the FG loops of mosquito-borne flavivirus EDIII are invariably longer than those of tick-borne flavivirus EDIII, which lack the RGD (Arg-Gly-Asp) motif. Inhibition of virus entry by peptides resembling the DENV-2 FG loop in mosquito cells *in vitro* first suggested its role in determining vector specificity [3]. Alignment of the amino acid sequences of mosquito-borne flaviviruses further demonstrated the RGD motif, especially the Arg and Gly residues, was largely conserved in the mosquito-borne flaviviruses in the JEV serocomplex, which utilize a substantially larger spectrum of host and vector species than other flaviviruses [4,5]. However, attenuated YFV 17D strains have the RGD motif while the virulent Asibi parental strain contains a Thr instead of Arg at position 380 [4]. These data highlight the need for understanding the function of the conserved Arg residue, which has been identified as a determinant of binding efficiency to negatively charged host molecules among flaviviruses [6]. Although characterization of the RGD motif has been performed in JEV, WNV, MVEV and YFV in mice, knowledge of its interaction with arthropod receptors remains limited [6-8]. Length of the corresponding region in DENV-2 EDIII played a more significant role than the sequence in infectivity, and substitution with the sequence of YFV 17D strain did not significantly decrease infectivity in *Aedes aegypti*. However, the sequence of YFV Asibi strain, which is more infectious and disseminates at higher rates in *Ae. aegypti*, was not evaluated [9-11]. More importantly, this model provided limited information on the RGD motif function due to the lack of conservation of this motif among the four DENV serotypes. Therefore, we utilized the YFV reverse genetics system to evaluate the impact of the Thr380Arg (T380R) mutation in EDIII on the viral infectivity and dissemination in *Ae. aegypti*. Such knowledge will increase understanding of the RGD-motif-dependent viral entry mechanisms among mosquito-borne flaviviruses utilizing different vectors.

Introduction of T380R mutation in E of infectious clones of YFV Asibi strain and 17D/Asibi M-E chimera was achieved by subcloning the structural genes into the pGEM®-T Easy vector (Promega, Madison, WI) followed by PCR site-directed mutagenesis with Quikchange®II XL kit (Stratagene, La Jolla, CA) and ligation of *BspE*I (New England Biolabs, Ipswich, MA)- and *Mlu*I (New England Biolabs)- digested plasmids as previously described [12]. Rescued viruses were obtained via electroporation of *in vitro* transcribed viral RNA in BHK-21 cells maintained in MEM-α media (Life technologies, Carlsbad, CA) supplemented with 10% FBS (Life technologies). Freshly harvested viruses mixed with defibrinated sheep blood were presented to 6 to 8-day-old *Ae. aegypti* RexD WE strain through a Hemotek membrane feeding apparatus (Discovery Workshop, Lancashire, England UK) at 37C for 60 minutes. Engorged mosquitoes were maintained with 10% sucrose solution at 16 hr:8 hr light–dark photoperiod for 14 days. Mosquitoes were cold anesthetized and dissected to collect the body and secondary tissues including the head, wings and legs of individual mosquitoes at 7, 10, and 14 days post infection (d.p.i.). The secondary tissues were used as the indicator for viral dissemination based on the disseminating phenotypes of the Asibi strain which causes the presence of infectious viruses in the head, wings and legs after the establishment of infection in the midgut [13]. Viral replication kinetics were assessed by titration of homogenized whole mosquitoes by TCID_50_ as described previously [10]. Infection was defined as the positive immunofluorescence staining in the serially-diluted homogenates of whole mosquito, body or secondary tissues in Vero cells. Dissemination was defined by the positive immunofluorescence staining in the homogenates derived from the secondary tissues in Vero cells. Fisher’s exact test was used to analyze the infection and dissemination rates and ANOVA was performed for the analysis of the titers of infected whole mosquitoes.

**Figure 1:**
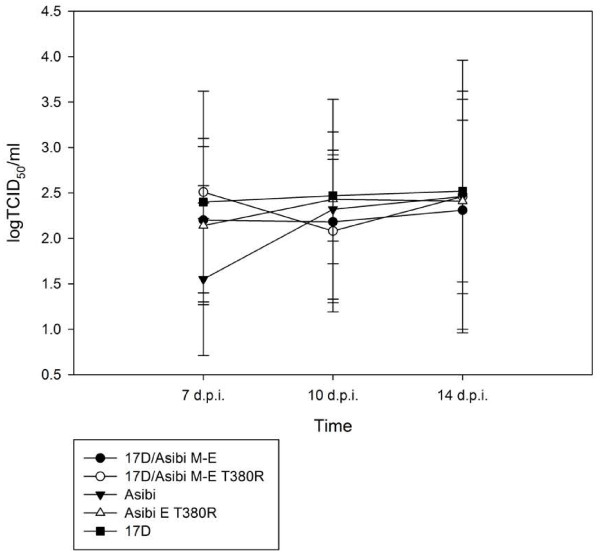
**Titers of whole-mosquito homogenates of *Ae. aegypti *** **at 7, 10 and 14 d.p.i.**

The infection and dissemination rates for all groups are summarized in Tables 1 and 2, respectively. The T380R mutation did not abolish viral infectivity, as similar infection rates of Asibi strain and Asibi E T380R mutant were observed at 7 (60.6% vs. 66.7%), 10 (50% vs.58.3%) and 14 (55.3% vs. 53.8%) d.p.i. Interestingly, a significantly higher infection rate was observed for the 17D/Asibi M-E E T380R mutant compared to the wild-type 17D/Asibi M-E chimera at 7 d.p.i. (91.3% vs. 41.4%,) and 14 d.p.i. (87.5% vs. 53.8%) (*p* < 0.05) although infection rates were not significantly higher at 10 d.p.i. The establishment of infection also led to similar dissemination rates both between the Asibi strain and Asibi E T380R mutant and between the 17D/Asibi M-E chimera and 17D/Asibi M-E E T380R mutant at 7, 10 and 14 d.p.i. Virus titers in whole mosquitoes were not significantly different for any group (Figure 1).

**Table 1:** ***Ae. aegypti *** **infection rates, calculated as number infected whole mosquitoes and bodies/tested**

	**Average blood meal titer (logTCID**_**50**_**/ml)**	**7 d.p.i.**		**10 d.p.i.**		**14 d.p.i.**	
Defibrinated sheep blood	N.A.	0/30	(0.0%)	0/24	(0.0%)	0/35	(0.0%)
17D	6.24	9/25	(36.0%)	8/12	(66.7%)	8/32	(25.0%)
17D/Asibi M-E	5.77	12/29	(41.4%)	13/24	(54.2%)	21/39	(53.8%)
17D/Asibi M-E E T380R	5.85	21/23	(91.3%)^†^	10/12	(83.3%)	14/16	(87.5%)^†^
Asibi	3.99	20/33	(60.6%)	9/18	(50.0%)	21/38	(52.6%)
Asibi E T380R	4.21	32/48	(66.7%)	21/36	(58.3%)	21/39	(53.8%)

^†^indicates the significant difference by Fisher’s exact test from 17D/Asibi M-E chimera.

**Table 2:** ***Ae. aegypti *** **dissemination rates calculated as positive secondary tissues/tested**

	**7 d.p.i.**		**10 d.p.i.**		**14 d.p.i.**	
17D	0/3	(0.0%)	1/5	(20.0%)	0/12	(0.0%)
17D/Asibi M-E	2/6	(33.3%)	3/8	(37.5%)	7/14	(50.0%)
17D/Asibi M-E E T380R	3/14	(21.4%)	1/6	(16.7%)	5/8	(62.5%)
Asibi	8/17	(47.1%)	4/5	(80.0%)	9/15	(60.0%)
Asibi E T380R	15/19	(79.0%)	9/14	(64.3%)	10/15	(66.7%)

Our results demonstrated the change in the biochemical properties between Thr and Arg did not reduce the viral infectivity and subsequent dissemination of YFV in *Ae. aegypti*. 17D/Asibi M-E E T380R mutant was able to infect a significantly higher percentage of mosquitoes at 7 and 14 d.p.i. than the 17D/Asibi M-E chimera, which was previously shown to have the lower infectivity due to the attenuation by the chimerization [12]. However, the results showed the likelihood of transmission should not be enhanced due to the similar dissemination rates between the wild-type and mutant viruses. The observation was partially consistent with the previously published study on DENV-2 based on the fact that the infectivity was maintained whilst the length of FG-loop was not altered. The more plausible explanation for the increased infectivity due to the T380R mutation may be that the gain of positive charge leads to the more efficient viral attachment to the target cells in the host and the higher infection rates. Although the increase of positive charges on viral structural proteins has been known to reduce the viral dissemination, most mutant viruses still retain the infectivity for susceptible host cells. The presence of the positive charges on the E2 glycoprotein of the mosquito-borne alphavirus eastern equine encephalitis virus led to the higher number of infected brain cells in mice intracerebrally challenged, indicating the presence of positively charged amino acids may be advantageous for the establishment of infection [14]. In the case of adeno-associated virus, the gain of positive charge through the similar Thr➔Arg mutation in the capsid region significantly increased the viral attachment to HeLa cells [15]. Our results suggest that the presence of the positively charged arginine on the FG loop may favor the establishment of infection in mosquitoes. Understanding the function of flavivirus RGD motifs and FG loops in viral infection and dissemination can only be achieved by further testing the hypothesis with the reverse genetics systems of *Culex*-borne flaviviruses, tick-borne flaviviruses and the newly discovered mosquito-only flaviviruses.

## Competing interests

The authors declare that they have no competing interests.

## Authors’ contributions

YSH constructed the viral mutants, maintained and infected mosquitoes, performed the virus titrations, conducted statistical analyses, and drafted the manuscript. JTN performed the mosquito infections. KMH participated in data analysis and interpretation and helped to draft the manuscript. ML conceived the study. DV and SH participated in study conception, design and coordination, and directed the research. All authors read and approved the final manuscript.
